# Designing the Sniper: Improving Targeted Human Cytolytic Fusion Proteins for Anti-Cancer Therapy via Molecular Simulation

**DOI:** 10.3390/biomedicines5010009

**Published:** 2017-02-17

**Authors:** Anna Bochicchio, Sandra Jordaan, Valeria Losasso, Shivan Chetty, Rodrigo Casasnovas Perera, Emiliano Ippoliti, Stefan Barth, Paolo Carloni

**Affiliations:** 1 German Research School for Simulation Sciences, Forschungszentrum Jülich, Jülich 52425, Germany; a.bochicchio@fz-juelich.de; 2 Computational Biomedicine, Institute for Advanced Simulation IAS-5 and Institute of Neuroscience and Medicine INM-9, Forschungszentrum Jülich, Jülich 52425, Germany; r.casasnovas.perera@fz-juelich.de (R.C.P.); e.ippoliti@fz-juelich.de (E.I.); 3 Department of Physics, Rheinisch-Westfälische Technische Hochschule Aachen, Aachen 52062, Germany; 4 Department of Integrative Biomedical Sciences, Institute for Infectious Disease and Molecular Medicine, University of Cape Town, Cape Town 7701, South Africa; sandrajordaanibms@gmail.com (S.J.); shivan.chetty@gmail.com (S.C.); 5 Scientific Computing Department, Science and Technology Facilities Council, Daresbury Laboratory, Warrington WA4 4AD, UK; valeria.losasso@stfc.ac.uk; 6 JARA–HPC, Jülich Supercomputing Centre, Forschungszentrum Jülich GmbH, Jülich 52425, Germany

**Keywords:** immunotherapy, targeted human cytolytic fusion proteins, molecular dynamics, high performance computing, Angiogenin, Granzyme B

## Abstract

Targeted human cytolytic fusion proteins (hCFPs) are humanized immunotoxins for selective treatment of different diseases including cancer. They are composed of a ligand specifically binding to target cells genetically linked to a human apoptosis-inducing enzyme. hCFPs target cancer cells via an antibody or derivative (scFv) specifically binding to e.g., tumor associated antigens (TAAs). After internalization and translocation of the enzyme from endocytosed endosomes, the human enzymes introduced into the cytosol are efficiently inducing apoptosis. Under in vivo conditions such enzymes are subject to tight regulation by native inhibitors in order to prevent inappropriate induction of cell death in healthy cells. Tumor cells are known to up-regulate these inhibitors as a survival mechanism resulting in escape of malignant cells from elimination by immune effector cells. Cytosolic inhibitors of Granzyme B and Angiogenin (Serpin P9 and RNH1, respectively), reduce the efficacy of hCFPs with these enzymes as effector domains, requiring detrimentally high doses in order to saturate inhibitor binding and rescue cytolytic activity. Variants of Granzyme B and Angiogenin might feature reduced affinity for their respective inhibitors, while retaining or even enhancing their catalytic activity. A powerful tool to design hCFPs mutants with improved potency is given by in silico methods. These include molecular dynamics (MD) simulations and enhanced sampling methods (ESM). MD and ESM allow predicting the enzyme-protein inhibitor binding stability and the associated conformational changes, provided that structural information is available. Such “high-resolution” detailed description enables the elucidation of interaction domains and the identification of sites where particular point mutations may modify those interactions. This review discusses recent advances in the use of MD and ESM for hCFP development from the viewpoints of scientists involved in both fields.

## 1. Introduction

Targeted cancer therapy has undergone extensive advancements in the past three decades. Immunotherapy, in particular, has shown much potential as a solution to the clinical challenges of conventional chemo- or radiation therapy such as systemic toxicity and drug resistance. Antibody-guided chimeric proteins, including antibody-drug conjugates (ADCs) and immunotoxins (ITs), have undergone several generations of optimization to improve their specificity and potency [1,2,3,4,5,6,7]. A number of ADCs (reviewed in [8]) and ITs (reviewed in [2,6]) have been undergoing clinical trials, with several already on the market due to their potential for disease-specific tumor suppression with much reduced side-effects compared with conventional therapies. However, drawbacks of earlier generation ITs, such as poor serum stability, immunogenicity [9], vascular leak syndrome [10] and up-regulation of tumor resistance mechanisms [11], have hampered their success for therapeutic applications. ITs comprising Pseudomonas exotoxin A (ETA) as effector domain have demonstrated potent cytotoxicity; however, due to their native ability to penetrate cells at will, they are also prone to off-target effects [12]. Developments towards entirely eradicating side-effects of ITs include removal of the native cell-penetrating domains of bacterial toxins (e.g., using truncated ETA’) [5] and depletion of B-cell and T-cell epitopes responsible for immunogenicity in response to non-human IT domains. The humanization of ITs has further elevated their therapeutic potential by rendering them less immunogenic than earlier generation ITs bearing non-human toxic or targeting moieties (e.g., ETA or mouse antibody) [13]. Human cytolytic fusion proteins (hCFPs) are a fourth generation class of ITs consisting of humanized antibodies or antibody derivatives, such as antigen-binding (Fab) or variable (Fv) antibody fragments [14,15], combined with a human cytotoxic effector. Human monoclonal antibodies and synthetic antibody derivatives (e.g., single chain variable fragment, scFv) are generated by means of transgenic mice, hybridoma or screening antibody/scFv phage display libraries [16,17]. Cell death effectors such as apoptosis-inducing enzymes (proteases, RNases or kinases) or other cytostatic proteins (e.g., microtubule-associated protein like MAP tau) are selected based on their cytotoxic activity [18,19,20,21,22,23,24,25,26,27], as well as their association with certain features of cancer, such as angiogenesis, accelerated cell division and immune system involvement. During hCFP generation, the pro-apoptotic protein and the scFv are expressed by means of a single construct, yielding a recombinant fusion protein with disease-specific tumor cell depletion activity.

On account of their apoptosis-inducing activity, cytolytic enzymes are subject to firm regulation by native inhibitors: a system intended to protect healthy tissues from inappropriate cell death. However, these protective pathways might be up-regulated during malignant cell transformation processes and become fundamental to cancer cell survival: many types of tumors possess an arsenal of cell death-escape mechanisms, rendering tumor cells resistant to apoptotic signaling [11]. Here we provide an overview of factors influencing the efficacy of enzymes, which have been used for hCFP formulation, as well as the molecular simulations methods that have been employed in order to circumvent these challenges and improve the efficacy of hCFPs for anti-cancer therapy.

### 1.1. Specific Enzymes Used in Human Cytolytic Fusion Proteins (hCFPs) Therapy and Their Inhibitors

Cytosolic inhibitors of apoptosis-inducing enzymes in target cells reduce the efficacy of hCFPs in malignant tumors, which putatively exhibit up-regulated expression of inhibitors. This causes a need for higher doses of hCFP treatment in order to saturate inhibitor binding and rescue enzymatic activity. Two enzymes, which have been tested to various extents for their specific cytotoxic activity in a range of cancer types, in cell culture and/or xenograft, are granzyme B (a serine protease) and angiogenin (human RNase A analogue). Both of these enzymes are limited for cytolytic efficacy by endogenous and frequently up-regulated inhibitors, however these limitations have been, to some extent and will be further, overcome by dry and wet lab methods to enhance their potential as hCFP effectors.

#### 1.1.1. Granzyme B

Granzyme B (GrB) is a serine protease critical for caspase 3-mediated apoptotic signaling. Tumor cells expressing tumor-associated or tumor-specific antigens induce an immune response, activating both B-cells (which produce antibodies against the tumor) and cytotoxic T lymphocytes (CTLs). The latter secrete cytotoxic granules, including GrB and perforin, in the vicinity of the tumor [22]. GrB is internalized by target cells, both by binding to a mannose-6-phosphate cell-surface receptor (in complex with serglycin) and by interaction with membrane-bound heparin sulfate [28]. Upon endocytosis, GrB escapes into the cytosol through endosomal membrane pores formed by inserted perforin. In the cytosol, GrB induces cell death by at least two pathways: the caspase-dependent apoptotic cascade or the caspase-independent mitochondrial pathway. Cleavage of caspase 3 by GrB serine protease degradation, directly after aspartic acid residues, triggers a cascade of caspase activation (cysteine proteases also cleaving at aspartate residues), culminating in caspase-dependent apoptosis. Alternatively, GrB cleaves Bid, which triggers mitochondrial cytochrome c release and loss of membrane integrity, resulting in a more necrotic form of cell death [29].

CTLs that produce granular GrB are protected for its cytolytic activity by an endogenous serine protease inhibitor 9 (Serpin 9 or PI9) [30,31]. This inhibitor has been shown to be up-regulated in some tumors, putatively providing an immune escape or pro-survival mechanism [32]. PI9 sequesters GrB by acting as a pseudo-substrate to which GrB binds irreversibly in a 1:1 stoichiometry, resulting in an inactive complex which is highly stabilized via salt bridges, hydrogen bonds and hydrophobic interactions. PI9 up-regulation therefore presents a considerable obstacle to immunotherapy whereby CTLs are activated to target malignancies, which thereby become insensitive to the cytotoxic granzyme B attack. Furthermore, inhibition by PI9 is the most potent native limiting factor to the cytolytic efficacy of hCFPs built on GrB pro-apoptotic activity [33].

#### 1.1.2. Angiogenin

Angiogenin (Ang) belongs to the RNase superfamily and is a human analogue of bovine RNase A. Ang has several seemingly contradictory functions in physiologically healthy tissues and in tumors [34]: via nuclear ribonuclease activity it is involved in signaling pathways promoting cell proliferation, angiogenesis, migration and invasion—all hallmarks of tumor progression. However, in the cytosol (where it is localized during conditions of oxidative stress) its ribonuclease activity results in detrimental tRNA degradation [35] and arrest of protein biosynthesis [18,19], ultimately resulting in apoptotic cell death. Wild type Ang exhibits relatively low ribonuclease activity in comparison with other RNases, attributed to endogenous regulation by the human placental ribonuclease inhibitor 1 (RNH1) which ensures suppression of cytosolic ribonucleolysis by binding to Ang with a 1:1 stoichiometry, reversibly but with high affinity [36,37]. Considering the cytolytic potential of Ang, tight regulation is certainly preferential for prevention of tissue damage, however, RNH1 also contributes to tumor resistance by inhibiting Ang’s stress-associated cytostatic activity and restricting its cytosolic translocation despite the high metabolic demands of rapidly-dividing malignant cells. Affinity of Ang for RNH1 is therefore a major limiting factor for the efficacy of hCFPs based on Ang as cytolytic effector and modulating this inhibition is crucial for the development of such targeted therapies [38,39].

## 2. Computational Methods for Protein Engineering of Relevance for This Review

Protein molecular diversity, structure and function are often studied by mutagenesis experiments. These techniques allow the generation of numerous protein mutants, some among which can have better activity than the wild types or even new different properties [40]. The inconvenience is that it is difficult to understand the connection between protein and function from these techniques [41].

Molecular simulation techniques accurately characterize interactions at atomic resolution. Hence, they can be used to design the structures, properties and functionalities of proteins in order to overcome the experimental drawbacks [42,43]. Several biocomputing methods (from state of the art homology modeling to molecular simulations) have been developed to predict structural determinants and extract functional insights from the large amount of sequence and structural data currently available. These cutting edge methods offer the possibility to (i) de novo design three-dimensional models of proteins with unknown structure; (ii) create models of protein-protein complexes and accurately characterize the interface residues, which are fundamental to the stability of those complexes. In particular, Molecular Dynamics (MD) simulations give precise description of the protein dynamics necessary to understand the underlying mechanisms of protein function. MD produces trajectories of the motions of atoms in time, providing direct insight into the molecular details of protein flexibility [44]. The latter is manifested on different length and time scales, ranging from fast bond and angle vibrations, to side chain rotamer flexibility and slower correlated local and global conformational motions. Protein fluctuations and transitions between substrates are known to be relevant for many biological processes, such as protein folding, enzyme catalysis, protein-protein/ligand interactions, signaling and allostery. MD coupled with enhanced sampling methods (ESM) has been successfully employed to describe these processes. In the following, we will report a short review of the computational methods used to design more potent hCFPs, based on GrB and Ang mutants as cytolytic effector domains. Other in silico methods, generally used in protein engineering, are described in Appendix A.

## 3. Proteins Structural Predictions

The most reliable strategy of protein structure prediction is arguably template-based bioinformatics modeling, also called homology modeling or comparative modeling [45].

Protein folds (i.e., the 3D arrangement and connectivity of secondary structure elements) turn out to be better conserved than protein sequences [46]. Proteins with similar sequences, even if distantly related, fold into similar structures and, furthermore, evolutionarily-related (homologous) proteins often retain the same three-dimensional fold despite the accumulation of divergent mutations and insertions or deletions in their sequence [47]. For pairs of distantly related proteins, i.e., those sharing a sequence identity of approximately 20%, the structural region with the same fold may comprise less than half of each molecule [48]. Hence, a “twilight zone” of 20%–35% sequence identity was defined, where it is not possible to unambiguously distinguish between protein pairs of similar and non-similar structures [49]. The quality of homology modeling-based predictions at the twilight zone may therefore vary widely [50]. These models may, however, be used to refine NMR structures, to find binding/active sites by 3D motif searching or to predict approximate biochemical functions [51]. When the sequence identity exceeds 30%, more reliable homology models can instead be constructed [52]. Highly reliable models do exist for sequence identities greater than 50% [45].

The homology modeling procedure essentially consists of four principal steps [51].

### 3.1. Template Search and Target-template Sequence Alignment

The procedure starts with the identification of evolutionarily related proteins serving as template(s) for modeling the structure of the target. Appropriate templates are usually identified by inquiring data bases of protein structures, (for example, the Protein Data Bank (PDB) [53]), with the target sequence as the query. The selection is then based on sequence alignments methods. Depending on the degree of sequence similarity, different algorithms can be used to perform sequence alignment. In the safe percentage identity zone (i.e., where sequence identity is greater than 30%), programs such as BLAST [54] or FASTA [55], which perform serial pairwise sequence alignments, are usually good enough to give a set of possible target homologs [55]. When the sequence identity to any experimental solved structure is very low, more sensitive methods based on comparative multiple sequence alignment (MSA), of which CLUSTALW [56] is one of the more commonly used, have been shown to give better results. These algorithms are capable of analyzing protein families and their evolution, as well as being able to detect remote homologs [57]. However, while the optimal solution to a pairwise alignment can be found within reasonable time, multiple sequence alignments are significantly more computationally demanding. Hence, a conventional approach is to do progressive alignment [58], which is an iterative pairwise alignment method that starts with the two closest sequences and progressively adds more distant ones. This allows dealing with a higher number of distantly related sequences; however, progressive methods are very dependent on the initial alignment and errors made at any stage of the multiple alignments are propagated through to the final result. Hence, iterative methods were proposed to improve on this weak point of the progressive methods. Iterative algorithms optimize a cost function (A cost function maps a value of one or more variables onto a real number intuitively representing some “cost” associated with the value) by assigning a score to an initial global alignment and then iteratively realigning sequence subsets and scoring these alignments. An extensive review of these methods is provided in [59]. Regardless of the chosen algorithm, the results of a multiple sequence alignment search in general contain useful information, such as the identification of conserved amino acids or similar amino acids groups with respect to their physico-chemical properties (i.e., aromaticity, aliphaticity, charge).

### 3.2. Template Selection

The best template(s) can be chosen according to different ways. These include (but they are not limited to) as described in [60]: (i) the selection of the template with the highest sequence similarity; (ii) the modeling of a theoretical template as average of other templates (ii) the unification of different templates for different regions to maximize the local similarity in each region; (iii) the derivation of constraints/restraints from templates and the successive construction of models that satisfy as many of these constraints as possible.

In addition, in choosing the template(s), it is important to take into account the accuracy and the condition of the experimental structures, such as resolution of X-ray-determined structures, the number of restraints per amino acids for nuclear magnetic resonance (NMR)-derived structures, the biological state of the protein (e.g., active or inactive) and the presence of bound ligands.

### 3.3. Model Construction

Three classes of model construction methods have been suggested [51]:

- Modeling by assembly of rigid bodies [61]. The model is assembled from a small number of rigid bodies obtained from aligned protein structures.
- Modeling by segment matching or coordinate reconstruction [62,63] Here one relies on the positions of conserved regions in the templates. A subset of atomic positions and short amino acid segments from template structures are used as a guide to build comparative models.
- Modeling by satisfaction of spatial restraints [64,65]. This method uses the structure of the templates to define restraints that are usually supplemented by stereochemical restraints on bond lengths, bond angles, dihedral angles, and non-bonded atom-atom contacts. The model is then derived by minimizing the violations of all the restraints in the aligned amino acids of the target sequence.

A variety of programs such as MODELLER[66], SWISS PDB Viewer [67], SCWRL [68] and web servers, such as 3D-JIGSAW [69] or SWISS-MODEL [70] allow to build structural models of the proteins based on target-template alignment. Regions of the target sequence that are not aligned to a template (gaps) are more difficult to predict. Insertions or deletions in the alignment are generally placed out of helices and strands, as well as within loops and turns, i.e., in the regions in which the template and the target may show quite different conformations [71]. There are two main approaches to gaps prediction: (i) Geometry optimization by an apt energy function; (ii) loops across the PDB [45] with endpoints that match those of the target structures are added.

### 3.4. Model Quality Assessment

Errors in these bioinformatics-based predictions may depend mostly on two connected factors:

- The sequence identity between target and template
- The sequence alignment

If the percentage of the sequence identity is 25% or lower, the alignment leads to very large errors [47]. Therefore, an essential step in the homology modeling process is the verification of the model. To assess the global quality of the model one can use indicators, which return a pseudo-energy score for the entire model. Common used global indicators include DFIRE [72], an all-atom distance-dependent statistical potential; QMEAN [73] a composite scoring function; DOPE [66], a statistical potential optimized for model assessment. A basic requirement of the predicted models is the stereochemical correctness. This may be evaluated using programs such as PROCHECK [74] and WHATCHECK [75]. The models may be validated through site-directed mutagenesis experiments, mass spectrometry, circular dichroism, cross-linking, fluorescence-based thermal shift, Förster Resonace Energy Transfer (FRET), light scattering or electron microscopy. In turn, as mentioned before, such experimental information can also be translated into constraints/restraints and introduced in the modeling protocols, thus improving the accuracy of the models. Bioinformatics crosschecks are also very useful: for instance, in the case of enzymes they can be used to confirm the position of important catalytic residues in the active site by comparison with homologous proteins [76].

## 4. Interface and Hot Spots Identification

Just a small number of residues typically account for more than three-quarters of the protein/protein binding free energy [77,78]. These residues, called hot-spots [77], are responsible for the stabilization of the protein/protein complexes and thus are interesting targets in protein engineering and drug design.

Experimental alanine-scanning mutagenesis is a potent method to analyze the occurrence of critical interactions at protein-protein interfaces. Single replacement of amino acids with alanine gives hints on the importance of the interactions at the interface: The method quantifies the effect of an amino acid side-chain deletion on the affinity of a protein-protein complex. While experimental alanine scanning still represents a large effort that cannot be applied easily to a large scale screening of protein–protein interfaces. Hence, a variety of computational alanine scanning protocols have been developed [79,80,81,82], allowing the automatic scanning of a complete protein-protein interface within minutes on a single PC processor. The Baker’s alanine scanning approach [79,80], implemented in the ROBETTA server [83], first defines the interface residues as (i) residues that have at least one atom within a 4 Å radius sphere of an atom belonging to the protein partner; or (ii) residues that result deeply buried upon complex formation [79,80]. The program then exchanges one by one the residues at the interface with alanines and computes the binding free energy for each mutant, allowing estimating the effect of the single mutation on the stability of the complex and the identification of potential hot spots. This free energy estimation relies on a simple physical model. It uses (1) all heavy atoms and polar hydrogen atoms to represent proteins and proposes a free energy function for linearly combining terms such as Lennard-Jones potentials to describe atomic packing interactions; (2) an orientation-dependent hydrogen bond potential derived from high-resolution protein structures [84]; (3) Coulomb electrostatics; and (4) an implicit solvation model [85]. The method can be combined with more sophisticated approaches for the calculation of changes in binding free energy upon alanine mutation, such as molecular mechanics-Poisson Boltzmann surface area (MM-PBSA) methods [86] and explicit solvent MD simulations, which are computationally much more expensive but can give a more accurate estimation of the effect of the alanine mutation in the proteins [87,88]. Machine learning algorithms have been also trained to predict hot-spot residues based on the use of physical/chemical data, as in K-FADE and K-CON [89]. For cases in which the 3D structure of a complex (or of a homologous one) is not available, very few hot-spot prediction methods can be used. One of them is the neural network-based method called ISIS [90].

## 5. Molecular Dynamics Simulations

MD simulations are an invaluable tool for investigating and understanding the underlying physical principles of structure and function of biological macromolecules [44]. They are used to calculate the dynamic behavior of a system by solving Newton’s equation of motion for the particles in the system as a function of time [91]. As such, conventional MD simulations involve three physical approximations: (1) decoupling of the motion of nuclei and electrons (Born-Oppenheimer approximation); (2) classical description of the motion of the nuclei; and (3) approximation of the quantum electronic interactions with an empirical potential energy function of the nuclear degrees of freedom (force field) [92]. In short, the time-evolution (trajectory) of a system with N particles and positions $R=\left\{R_{1},\ldots ,R_{N}\right\}$ is calculated at each time step by:

(1) $$m_{i}\frac{\partial^{2}R_{i}}{\partial t^{2}}=-\nabla_{R}\;V\left(R\right),\;i=1,\ldots ,N$$

which corresponds to the second Newton’s law. Variables $m_{i}$ and $R_{i}$ denote the mass and the position of particle i, respectively. V(R) is the potential energy of the system approximated by an empirical potential energy function of the form:

(2) $$\begin{array}{c} V\left(R\right)=V_{b}+V_{a}+V_{\mathrm{dih}}+V_{\mathrm{imp}.\mathrm{dih}}+V_{\mathrm{LJ}}+V_{\mathrm{coul}}\\ \;=\underset{\mathrm{bonds}i}{\sum }\;\frac{k_{i}}{2}{\left(l_{i}-l_{i,0}\right)}^{2}+\underset{\mathrm{angles}i}{\sum }\;\frac{f_{i}}{2}{\left(\varphi_{i}-\varphi_{i,0}\right)}^{2}\\ \;+\underset{\mathrm{dihedrals}i}{\sum }\;\frac{V_{i}}{2}\left[1+\cos\left({n\phi }_{i}-\phi_{i,0}\right)\right]+\underset{\mathrm{imp}.\mathrm{dih}.i}{\sum }\;k_{i}{\left(\xi_{i}-\xi_{i,0}\right)}^{2}\\ \;+\underset{\mathrm{pairs}i,j}{\sum }\;4\varepsilon_{i,j}\left[{\left(\frac{\sigma_{i,j}}{r_{i,j}}\right)}^{12}-{\left(\frac{\sigma_{i,j}}{r_{i,j}}\right)}^{6}\right]+\frac{q_{i}q_{j}}{4\pi \varepsilon_{0}\varepsilon_{r}r_{i,j}} \end{array}$$

Here, the first four terms represent the bonded interactions: the bond potential $V_{b}$, the bond angle potential $V_{a}$, and the improper dihedral potential $V_{\mathrm{imp}.\mathrm{dih}}$ (describing out-of-plane distortions), which are modeled as harmonic potentials, and the proper dihedral potential $V_{\mathrm{dih}}$, which is modeled by a sinusoidal term. The last two terms describe the pair-wise non-bonded interactions. The short-range repulsive and attractive dispersion interactions are typically represented by a Lennard-Jones (LJ) potential, where the parameters $\sigma_{ij}$ and $\varepsilon_{ij}$ shape the width and strength of the potential. The electrostatic interactions are represented by the Coulomb term, where $q_{i}$ denotes the partial charge of particle i, and the relative dielectric constant $\varepsilon_{r}$ is typically set to 1. Widely used force fields (FFs) include AMBER [93], CHARMM[94], and OPLS [95]. These have attained such a high standard of quality that the preference for one over the other is often dictated by practical considerations only, related to their implementation with the MD engine of choice. The calculation of the long-range non-bonded interactions impacts significantly on the computational cost of the simulation. It requires a sum of pairs of atoms, meaning it scales quadratically with the number of particles N in the system. To avoid this, LJ interactions are usually cut off above 1.0–1.4 nm [96]. Coulomb interactions, on the other hand, cannot simply be cut off due the long-range nature of the Coulomb potential that decays slowly, with only *r*^−1^ (see Equation (2)). Therefore, long-range electrostatic interactions are usually treated with the so-called “particle-mesh Ewald (PME) method” [97,98], which allows for NlogN, instead of *N*^2^, scaling with the number of particles *N*. Equation (1) can be analytically solved only for a few atoms. For biological macromolecule simulations, numerical methods must be used to split the integration of the equations of motion into discrete time intervals or time-steps *δt*. The velocity Verlet is a simple and widely used integration algorithm in MD codes. Within such an integrator, the velocities and positions of the particles at each time step are computed as:

(3) $$\begin{array}{c} r\left(t+\delta t\right)=r\left(t\right)+v\left(t\right)\delta t+\frac{F\left(t\right)}{2m}\delta t^{2}\\ v\left(t+\delta t\right)=v\left(t\right)+\frac{F\left(t+\delta t\right)+F\left(t\right)}{2m}\delta t \end{array}$$

The time step *δt* needs to be smaller than the fastest motions in the system, in order to prevent integration errors. However, not all vibrations need to be explicitly modeled to achieve a realistic description of the system, which enables the usage of a larger time step and renders the computations more efficient. Namely, bond vibrations are in their quantum ground state and are therefore better represented by a constraint, rather than a harmonic potential [99]. Constraining bond lengths allows increase of the time step to 2 fs. Widely used constraint algorithms are SETTLE [100] (for the water molecules) and LINCS [101] (for the rest of the system). The next fastest oscillations are given by the bond angles of hydrogen atoms that are usually important to be correctly described because related to the hydrogen bond network. Newtonian dynamics allows one to sample a statistical ensemble of microstates characterized by a constant number of particles (*N*), volume (*V*), and energy (microcanonical ensemble).

In order to reproduce typical experimental conditions, it is possible to control the system’s temperature and pressure during the simulation. Constant temperature is maintained through algorithms called thermostats [102] that allow fluctuations in total energy as to sample a canonical ensemble at thermodynamic equilibrium during the MD evolution. The system temperature is forced to attain, on average, the desired macroscopic value through proper alterations of the equations of motion. Very similar considerations apply for the so-called barostat algorithms [103], by which the pressure is controlled by opportunely scaling (isotropically or non-isotropically) the system volume (isothermal–isobaric ensemble). By using these algorithms and exploiting HPC resources that modern supercomputing centers can provide, MD simulations currently can simulate the evolution of a protein in realistic conditions for hundreds of microseconds and more.

A statistical mechanics-based analysis of MD trajectories allows quantitative estimates of important thermodynamic observables. The coordinates of a system formed by N atoms define a 3N dimensional configurational space and the coordinates plus momenta define a (3 + 3)N dimensional space called the phase space *Γ*. Each point of the phase space corresponds to a state of the system for which every property *O* takes a particular value *O*(*Γ*). In experiments, the system is defined under certain conditions like temperature and pressure. These conditions constrain the system to a subset of points in *Γ* that we call ensemble. Then, the observable *O*_obs_ of the property *O* is given by the average of *O*(*Γ*) over the states in the ensemble <*O*_ens_>. The system visits the states of the ensemble with a particular probability distribution p(Γ), which can be used to calculate the ensemble (i.e., thermodynamic) average as:

(4) $$O_{\mathrm{obs}}={\langle O\rangle }_{\mathrm{ens}}=\int O\left(\Gamma \right)p\left(\Gamma \right)d\Gamma$$

where $d\Gamma =dr_{1},\ldots dr_{N},dp_{1},\ldots dp_{N}$. The probability distribution (i.e., the specific ensemble under consideration) depends on the macroscopic parameters (like the number of particles *N*, the temperature *T* or the pressure *P*) that define the experimental conditions. In the case where *N*, *V* and *T* are kept constant (canonical ensemble), the corresponding probability distribution at thermodynamic equilibrium is proportional to the Boltzmann distribution function

(5) $$p_{NVT}=\frac{\exp\left[-\frac{V\left(\Gamma \right)}{k_{B}T}\right]}{Z}$$

where

(6) $$Z=\int d\Gamma p_{NVT}\left(\Gamma \right)=\int d\Gamma \exp\left[-\frac{V\left(\Gamma \right)}{k_{B}T}\right]$$

is the classical configuration integral, $k_{B}$ is the Boltzmann constant and V(Γ) is the value in Γ of the potential energy *V* of the system.

Molecular Dynamics is a powerful technique for the calculation of ensemble averages. MD simulates the time evolution of the system in the phase space in a particular ensemble. Starting from given initial coordinates and momenta Γ(0), the equations of motion are integrated over time *t* in the interval [0, τ] to generate a trajectory Γ(t). Then, the ensemble average ${\langle O\rangle }_{\mathrm{ens}}$ is approximated from the average over the trajectory ${\langle O\rangle }_{\tau }$ as:

(7) $${\langle O\rangle }_{\tau }=\frac{1}{\tau }\int_{0}^{\tau }\;O\left[\Gamma \left(t\right)\right]dt$$

If the system is ergordic the averages (4) and (7) will coincide in the limit t→∞.

(8) $$\lim_{\tau \to \infty }{\langle O\left(\Gamma \right)\rangle }_{\tau }={\langle O\left(\Gamma \right)\rangle }_{\Gamma }$$

Even if this cannot be mathematically proved for the most of the systems, it is usually assumed that all the physical systems have this property [104]. This is known as the ergodic hypothesis and is the theoretical justification for employing MD simulations as a tool to calculate ensemble averages of molecular systems.

It is crucial to acknowledge the limitations of MD in order to make optimal use of it. Commonly used FFs for proteins are approximate and they lack charge polarizability. To overcome this limitation, polarizable FFs have been developed, which can mimic, to a certain extent, the electronic redistribution in response to an external electric field [105]. Polarizable FFs are very promising[106]; however, they remain quite computationally demanding, with their use and parametrization being less user-friendly than that of their fixed-charge counterparts. FF cannot be used to study chemical reactivity, since chemical bonds cannot be broken or formed during MD. In addition, biological processes involving macromolecular complexes take place over a wide variety of time scales, spanning from hundreds of picoseconds to seconds or minutes. Presently, the advance of high-performance computers [107], special-purpose hardware dedicated to MD as ANTON [108,109] and the increase of the efficiency of the MD software [110], allow for simulation lengths in the microsecond to millisecond. Enhanced sampling methods (ESM) that allow one to effectively “accelerate” MD so as to sample more efficiently the relevant part of the phase space and be able to study interesting problems with affordable computational effort. Some of these methods are summarized in Appendix A; the next section provides an overall view of the Replica Exchange method. This method has been successfully employed to folding problems [111] and protein design [112], including one of the examples reported later.

## 6. Replica Exchange MD Simulations

Biological processes often occur in environments with free energy surfaces characterized by many minima. Barriers between minima may be difficult to cross at physiological temperature in the time scale accessible by the simulation. In these conditions, MD results strongly depend on the initial conditions that dictate the portion of the phase space region explored by the simulation.

Replica Exchange Molecular Dynamics simulations (REMD) enhances the sampling by running numerous independent copies (or replicas) in slightly different ensembles. Then, an exchange of the coordinates of the replicas is periodically attempted. The central idea is that one replica is under the desired conditions (e.g., the ensemble at 300 K and 1 atm), while others are unphysical but can access remote parts of the phase space. Through the occasional exchanges, the “good” replica then gets occasionally transported to different basins that it would never have visited due to barriers in between.

It can be proven that, if (i) valid ensembles are produced by the replicas’ exchange (ii) the exchange probability is high enough and produces good mixing over the ensembles, then, the sampling will be correct. In particular, the probability of a replica being in a state *x* depends on the potential energy *V* and the temperature *T* according to:

(9) $$p\left(x\right)\propto \exp\left[-\frac{V\left(x\right)}{k_{B}T}\right]$$

If the distribution of the energy states of two ensembles significantly overlap means that when a state is observed in one ensemble there is appreciable chance that the same state belongs to the other one as well. The exchanging attempts can be performed through a Metropolis-style Monte Carlo move based on the energy differences of the states. If the attempt is successful, the coordinates of the replicas are exchanged. In this way the so-called “detailed balance condition” is achieved and the sampling in the ensembles is correct independently of the occurrence of the exchange.

In a temperature replica-exchange simulation (T-REMD) or parallel tempering [113] one performs the same simulation over a range of temperatures. This class of methods relies on the fact that in an Arrhenius process the probability of crossing an energy barrier increases exponentially with temperature [103]. Since trajectories at higher temperatures are more likely to cross free energy barriers, leading to a wider array of sampled configurations, and they can filter down to lower temperatures, the ergodicity of the sampling at the lower temperature is enhanced.

Whereas T-REMD is a powerful method and it has been applied to practically all biochemical systems with great results, temperature is an intensive quantity and does not allow the selective enhancement of specific degrees of freedom that often are known to be the relevant ones in the process of interest and one would exploit this information in order to be more effective.

In this context, scaling portions of the system Hamiltonian is a common approach (H-REMD), in order to find better convergence behavior, especially in the case of large systems. T-REMD and H-REMD can also be combined, by integrating them on each replica [114] or in a multidimensional framework [115]. A promising H-REMD technique, especially in sampling biological molecules in explicit solvent is the Replica-Exchange with Solute Tempering (REST) [116]. In this method solute-solvent interactions in the Hamiltonian are modified so that only the solute biomolecule is effectively heated up while the solvent remains cold in higher temperature replicas. This way, the number of the replicas required is greatly reduced. In fact, the number of replicas needed to get efficient sampling in T-REMD scales as *d*^1/2^, where d is the number of degrees of freedom of the whole system (this is the main factor that limits the applicability of T-REMD for large systems). In contrast, it has been shown [116] that the required number of replicas in REST scales as (*d*_p_)^1/2^, where *d*_p_ is the number of degrees of freedom of the solute. The REST method is implemented by scaling specific force-field parameters of the solute differently in each replica, making the acceptance probability for the exchange of replica configurations independent of the number of solvent molecules in the system. This helps improving the scaling, reducing the number of required replicas and consequently the computational load [116]. It has been found [117] that when REST is applied to large systems involving large conformational changes, it can be less efficient than REMD. For example, it could happen that the lower temperature replicas stay in the folded structure while the higher temperature replicas stay in the extended structure: in this case, the exchange between those two conformations could be very low. In order to sample the folded and unfolded conformations of proteins more efficiently, the REST algorithm has recently been refined by modifying the original Hamiltonian scaling procedure. This is the Replica Exchange with Solute Scaling or REST2 approach [118] that has been employed in one of the examples presented later.

## 7. Results: In Silico and In Vitro Studies on Granzyme B and Angiogenin

### 7.1. Granzyme B

**Figure 1:**
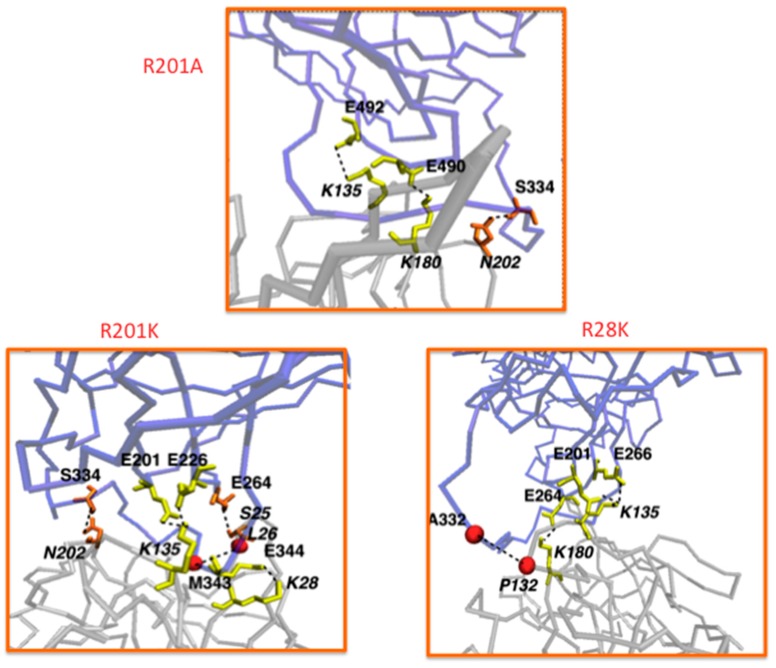
Contacts (yellow = salt bridges; orange = hydrogen bonds; red = hydrophobic interactions) left at the interface between PI9 (violet) and GrB (silver) after 50 ns MD simulation. Top = GrB mutant R201A; bottom left = R201K; bottom right = R201K.

Recombinant ITs based on human GrB demonstrated a comparable cytotoxicity with bacterial toxins both in vitro [23,119] and ex vivo [23]. Their human origin negates the dose-limiting side effects and the induction of neutralizing antibodies in patients, as reported in bacteria- or plant-derived chimeric ITs [120,121]. However, GrB enzymatic activity is shut down by the cytosolic expression of its natural inhibitor Serpin B9 (PI9), thus preventing a cell from apoptosis. PI9 is mainly expressed in cytotoxic T lymphocytes and natural killer cells. However, several types of tumor cells also produce this inhibitor in order to protect against the immune mediated killing [122]. PI9 first forms a reversible Michaelis-like complex with GrB and then covalently binds GrB with a stoichiometric ratio of 1:1 [123]. Here, a highly flexible PI9 peptide loop, known as the reactive center loop (RCL), is cleaved at the peptide bond between the so called P1 and P1’ residues [124]. The interaction with GrB via an ester bond at P1 [125] results in a dramatic conformational change where the RCL is inserted as a middle strand in the central β-sheet, thereby distorting and inactivating GrB [126]. Therefore, the design of GrB variants able to retain their cytotoxic activity, while binding more weakly to the PI9 inhibitor, would remarkably improve GrB therapeutic potential. Mutated residues have to be located at the GrB-PI9 binding interface but far enough from GrB active site. This requires the knowledge of the molecular details of the GrB-PI9 non-covalent or covalent interaction, which are unfortunately not available. Hence, the structure of the GrB-PI9 Michaelis complex (Figure 1) were predicted using computational tools, starting from the rat trypsin-*Manduca sexta* serpin B1 complex (PDB 1K9O) [33]. The two proteins are functionally related; they have similar length and good sequence identity (36% and 27%, respectively) to GrB and PI9. Moreover, human GrB structure is known (PDB 1IAU) and is structurally similar to rat trypsin (backbone RMSD = 0.6 Å). The active site of serpins is also known to be structurally conserved [127]. The computational procedure thus involved the following steps: (1) homology modeling of human PI9 based on *M. sexta* serpin B1; (2) structural fitting of human GrB onto rat trypsin; (3) structural fitting of modeled human PI9 onto *M. sexta* serpin B1; (4) refinement of the derived complex via 30 ns-long molecular dynamics simulation. To identify crucial interactions for GrB-PI9 binding, the computational alanine scanning method was applied to the optimized complex. The largest ΔΔG values turned out to be associated with the GrB mutants K27A, R28A and R201A. Therefore, these mutations were applied to the refined GrB-PI9 wild-type complex, together with the double mutant R28A-R201A and the related variants R28E, R28K, R201E and R201K. All the mutant complexes were simulated for 50 ns. In order to monitor the destabilization of GrB-PI9 mutant complexes over time, the following quantities were analyzed: (1) formation/disruption of salt bridges, hydrogen bonds and hydrophobic interactions at the protein-protein interface; (2) variation of distance between the GrB and PI9 mass centers; (3) root mean square deviation (RMSD); (4) variation of solvent accessible surface area (SASA) of the complex.

**Figure 2:**
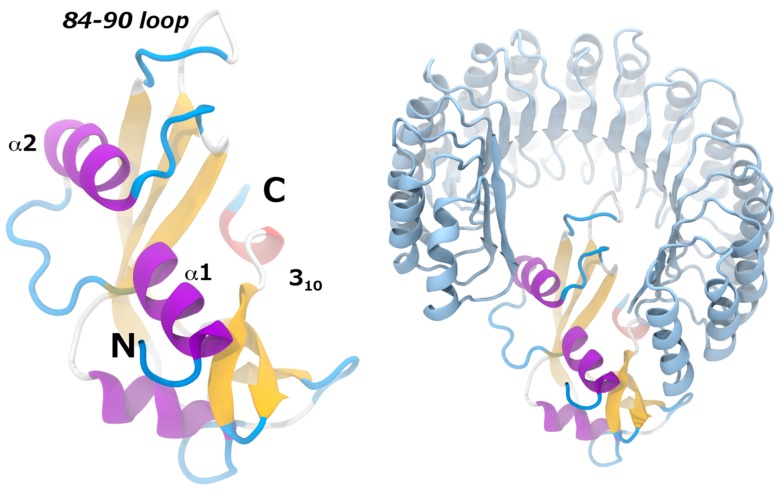
Left, Cartoon representation of the secondary structure of angiogenin. α-helices, β-strands, 3_10_ helices and loops are shown respectively in purple, yellow, blue and red. The N and C labels indicate the position of angiogenin N- and C- terminals. Labeled helices α1, α2, 3_10_ and the loop comprising residues 84–90 form important interactions with the RNH1 inhibitor. Right, cartoon representation of the angiogenin-RNH1 complex from X-ray experiments (PDB code 1A4Y).

Based on this analysis, it was predicted that the GrB mutations R201A, R201K and R28K are the most disruptive of the GrB-PI9 binding interface (Figure 2). The mutated residues are located in solvent exposed regions distant from the active site; hence their enzymatic activity was expected to be not too dissimilar from that of the wild type. This hypothesis was validated by performing enzymatic assays in vitro. The enzymatic activity of the GrB mutants in presence or absence of PI9 was measured. Interestingly, it was found that the most destabilized mutants R201A, R201K and R28K show a catalytic activity similar to that of wild-type GrB in the absence of PI9, but also still significant (46% to 94%) in presence of PI9. Strikingly, the R201K mutant retained a very high activity (94%) even after incubation with high concentration of PI9. GrB R201K insensitivity to its natural inhibitor, which is often expressed in solid tumors, makes it a very promising lead candidate for immunotherapy against various cancer types [33]. Moreover, a GrB variant that can kill both PI-9 positive and PI-9 negative tumor cells will provide a significant advantage for the treatment of relapsing tumors [128].

### 7.2. Under Special Consideration: GBR201K as a Tool to Improve Other Immunotherapeutic Strategies Aiming to Attack Serpin B9 Positive Tumor Cells

The encouraging in vitro data on GrB mutants, especially R201K, motivated the generation of modified GrB-based hCFPs for the treatment of different types (solid or hematologic) of tumor malignancies. Several tumors have indeed been described to express PI9, such as breast cancer [129], metastasizing melanoma [130], lung cancer [131] and prostate cancer [132].

#### 7.2.1. hCFPs Against Liquid Tumors: Chronic and Acute Myelomonocytic Leukemia

Chronic myelomonocytic leukemia (CMML) is a hematopoietic malignancy characterized by peripheral blood monocytosis. It progresses in a third of cases to acute myelomonocytic leukemia (AMML), a quickly developing malignant proliferation of myeloblasts [133]. The three most promising granzyme B mutants (GrBR28K, GrBR201A and GrBR201K) were used paired with the antibody fragment H22(scFv) to generate hCFPs specifically targeting the surface antigen CD64. It was shown that CD64 is expressed on the majority of CMML and AMML cells, making CD64 a potential target for CMML and AMML treatment. The hCFPs based on both wild-type and R201K-mutated GrB were tested and it was found out that the two variants are equally effective in killing cell lines not expressing PI9, but Gb(R201K)-H22(scFv) is more toxic against CMML and AMML cells with up-regulated PI9. Interestingly, it also showed higher activity to some AMML and CMML probes less sensitive to Pseudomonas exotoxin A. These results suggest that mutant GrB-based hCFPs targeting CD64 show a great potential for a specific improved immunotherapy against CMML and AMML [134].

#### 7.2.2. hCFPs Against Solid Tumors: Hodgkin Lymphoma, Rhabdomyosarcoma, Triple-Negative Breast Cancer

Classical Hodgkin lymphoma (cHL) is the most common subtype of Hodgkin lymphoma, characterized by abnormal Reed-Sternberg cells in the peripheral blood. cHL was characterized to express PI9, providing resistance to immunotherapy treatment [135]. To address this issue, Schiffer et al. [136] fused the R201K-mutated GrB to the Ki4(scFv) fragment specifically targeting the cHL-selective receptor CD30. The GrBR201K-H22(scFv) construct was used as unspecific control. No difference in cytotoxic activity between GrB-Ki4(scFv) and its mutant R201K was detected on PI9-negative cells in vitro, but only the mutant was able to kill both PI9-positive and negative tumor cells in vitro as well as in vivo*.* These results support the therapeutic potential of mutant GrB-based Ki4(scFv)-derived constructs for the treatment of cHL as well as other PI9-positive resistant tumors [136]. A novel hCFP composed of the R201K-mutated GrB and a human fragment scFv1711 derived from Panitumumab was also engineered. This scFv has already been exploited for the design of third generation immunotoxins and is derived from an approved human antibody binding to epidermal growth factor receptor (EGFR), which is overexpressed in several human cancers including the rare childhood cancer rhabdomyosarcoma (RMS), but rarely expressed in surrounding healthy cells [137]. The new hCFP (E)GrBR201K-scFv1711 demonstrated selective binding and potent pro-apoptotic effects at nanomolar concentrations against epidermoid cancer cells, as well as against rhabdomyosarcoma cells in presence or absence of the endosomal disrupting agent chloroquine, making it a promising novel therapeutic agent against several types of solid tumors [138].

Another hCFP, GrBR201K-αEpCAM(scFv), was designed by combining with the mutant R201K-GrB an antibody fragment against the epithelial cell adhesion molecule (EpCAM), a transmembrane glycoprotein mediating epithelial cell-cell adhesion which is expressed in triple negative breast cancers (TNBC) associated with an unfavorable prognosis [139]. The construct showed specific binding, internalization and ability to inhibit growth of TNBC cells in vitro and to induce apoptotic pathways through the activation of caspase 9. The tumor site-specific targeting and toxicity of GrBR201K-αEpCAM(scFv) were also confirmed in vivo, suggesting a great potential for the treatment of TNBCs [26].

#### 7.2.3. Other Applications

The antimalarial activity of GrB-based hCFPs by combining GrBR201K with 2.44IgG1, a murine antibody specifically targeting the merozoite surface protein MSP4, was tested in vitro. This protein includes strain-specific (EGF)-like domains and is imported into newly infected erythrocytes without significant processing, making it an attractive target for antibody-guided immunotoxins. The fusion construct was shown to be able to inhibit Plasmodium falciparum growth through internalization and activation of the metacaspase MCA-1 or induced eryptosis in infected erythrocytes [140,141]. This study confirms the therapeutic potential of GrB-based recombinant antibody mediated immunotherapeutics even against non-cancer diseases [142].

### 7.3. Angiogenin

The human ribonuclease angiogenin (Ang) has been shown to be a promising candidate for the effector domain of hCFPs because of its ribonucleolytic activity [143,144,21]. However, the application of Ang-based hCFPs is hindered by RNH1, which is present in high concentration in all human cell types (i.e., more than 0.01% of total intracellular proteins) and AngWT has a particularly high affinity for RNH1 (in the femtomolar range) [36]. While Ang is found primarily in the nucleus during growth conditions, RNH1 is found more abundantly in the cytoplasm and has been shown to reduce cytoplasmic AngWT activity, while nuclear Ang remains enzymatically active [37]. Since Ang-based hCFPs rely on cytoplasmic deployment of apoptosis-inducing Ang, target tumor cells can protect themselves by expressing inhibitor proteins such as RNH1 and rapidly neutralizing wild-type (WT) Ang. A possible strategy to increase the efficacy of Ang-hCFPs-based therapies is to design Ang-hCFPs mutants with reduced affinity for the RNH1 inhibitor. In the following example, experiments and molecular simulations were combined in pursuit of this aim. Cytoplasmic inactivation of AngWT reduces or abolishes hCFP efficacy, thus desensitizing Ang-hCFPs to inhibition is crucial for retention of their cytotoxicity. Ang residues 85 and 86 have been identified as residing in the interacting region for RNH1 [36], while residue 117 resides in the enzymatic active center of Ang [36]. Combining an inhibition-desensitizing mutation with further mutation(s), which increase enzymatic activity, offers a promising strategy for enhancing hCFP potency.

Recently, Cremer et al. [39] developed Ang mutants that were either more active or showed less affinity for RNH1 than AngWT. In particular, Ang Q117G and Ang G85R/G86R mutations (QG_mut_ and GGRR_mut_ hereafter) showed higher enzymatic activity and less affinity for RNH1 than AngWT, respectively. However, these two mutations do not act synergistically. In fact, the enzymatic activity of the combination mutant GGRR/QG_mut_ is similar to that of QG_mut_ but its affinity for RNH1 was comparable to that of AngWT. Therefore, although the combination of the two mutations resulted in Ang mutants with better ribonucleolytic activity, the affinity for the RNH1 inhibitor was restored to AngWT levels [39]. Then, molecular dynamics simulations were performed to understand how mutations of Ang impact the affinity for RNH1 [38]. Based on this knowledge, new mutants with high ribonucleolytic activity and low RNH1 affinity could eventually be generated.

**Figure 3:**
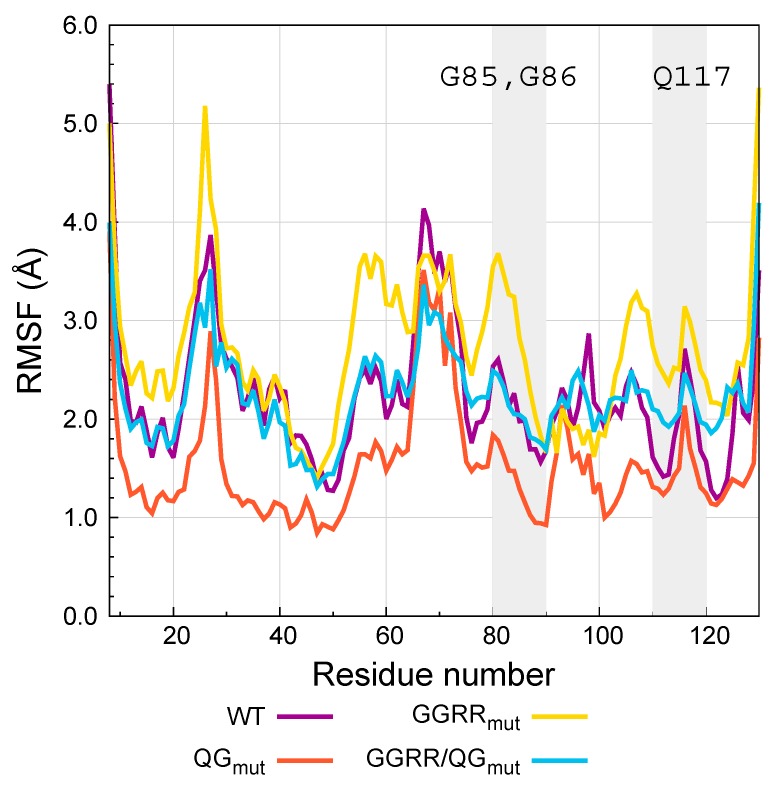
Root mean square fluctuations (RMSF) of the C_α_ carbons of WT angiogenin and the mutants QG_mut_, GGRR_mut_, GGRR/QG_mut_ complexed with RNH1 measured from REST2 simulations. The grey-shaded areas highlight the regions neighboring the G85R, G86R and Q117G mutations.

For the molecular dynamics study, Cong et al. [38] prepared the RNH1 complexes with AngWT and with the mutants GGRR_mut_, QG_mut_ and GGRR/QG_mut_ in aqueous solution mimicking the temperature and ionic strength conditions of the in vitro experiments. The complexes of RNH1 with AngWT and the mutants were built based on the X-ray structure of the RNH1-AngWT (PDB code 1A4Y) using the SWISS PDB Viewer. The N- and C-terminals of the hCFPs linkers present in the experiments were also modeled on the Ang structures. As explained above, standard MD simulations suffer from limitations in sampling the large timescales on which protein-protein association/dissociation take place. Therefore, in this cases enhanced sampling methods are necessary to understand the interaction between RNH1 and the Ang variants. Among the many possible enhanced sampling approaches, the ones requiring the definition of a reaction coordinate or CVs on which the sampling is enhanced are not optimal to study highly multidimensional processes such as protein-protein association/dissociation. This because proteins are composed of hundreds of atoms, and the number of relevant degrees of freedom needed to describe in full detail protein-protein association/dissociation processes is enormous when using atomic resolution. Furthermore, not a lesser number of solvent molecules participate in these processes, which would further increase the number of important degrees of freedom that should be appropriately described in the CVs. In Cong et al.’s work, Replica Exchange with Solute Tempering (REST2) simulations were employed for all the RNH1-Ang complexes. In fact, temperature-based Replica Exchange methods (see Replica Exchange simulations section) provide a way to enhance the fluctuations of all the degrees of freedom of all the particles in the system, thus allowing unbiased sampling of highly multidimensional processes. In particular, in the REST2 formulation the algorithm is applied only to a subset of atoms in the system, typically the “solute”—the two proteins in the present case—and not to the solvent molecules. Through the REST2 simulations, the most stable conformations of the RNH1-Ang complexes could be identified. This information, in turn, allowed a more in-depth understanding of the protein-protein interactions involved in each case, and the flexibility of each complex, which was correlated to the affinities of the Ang variants to RNH1 observed experimentally. In all cases, the RNH1 accommodates the Ang protein in the same mode, in which Ang binds in the center of the RNH1 horseshoe-shaped structure (Figure 3). Helices α1, α2 and 3_10_, together with the loop formed by residues 84–89, form the main interactions with the RNH1 inhibitor (Figure 3). In particular, in the RNH1-AngWT complex G85 and G86 interact with solvent molecules. G86 also interacts with Q346 and S289 of RNH1. The other relevant mutation affects Q117, in the 3_10_ helix. In the RNH1-AngWT complex, this residue interacts with solvent molecules and with F120, R121, T44 and I42 of AngWT and with D435 of RNH1. The RNH1-QG_mut_ complex shows similar interactions in the loop containing G85 and G86, however strengthened intermolecular interactions were observed between angiogenin residues as well as strengthened association between the AngQG_mut_ with RNH1. The mutation of Q117 had also longer-range effects stabilizing the α2-helix interactions with RNH1. The mutations G85R and G86R change the interactions of the 84–90 loop with RNH1, as R85 and R86 form salt bridges with E149, E206 and E264 of RNH1 in the RNH1-GGRR_mut_ complex. These mutations have long-range effects on helix α2, whose interactions with RNH1 are weakened, and Q117, which swings to a solvent-exposed conformation in the RNH1-GGRR_mut_ complex. The interactions of the mutated R85 and R86 are weakened in the RNH1-GGRR/QG_mut_ complex, but, interestingly, the angiogenin-RNH1 interactions of helices α2 and 3_10_ are recovered in the complex with the GGRR/QG_mut_ mutant, where G117 forms salt-bridge interactions with RNH1.

In order to understand the impact of the mutations on the flexibility of the complexed proteins, two physical quantities, the root mean square deviation (RMSD) and the root mean square fluctuations (RMSF), were calculated from the equilibrium simulation trajectory of the (biologically relevant) replica at 310 K.

The RMSD is defined as

(10) $$\mathrm{RMSD}=\sqrt{\frac{\sum_{i=1}^{N}{\left(r_{i}-r_{i\_ref}\right)}^{2}}{N}}$$

where *r_i_* is the position of atom *i*, *r_i_ref_* is the position of the same atom in a reference structure and N is the total number of atoms considered in the calculation of the RMSD. Therefore, this quantity provides a measure of the average distance of a set of atoms in the protein with respect to the same atoms in the reference structure. In the present case, the RMSDs were calculated for all the complexes by taking only into account the C_α_ atoms of the RNH1 and the Ang variants. In this way, the RMSDs of mutants can be directly compared. The reference structure was in all the cases the X-ray structure of the RNH1-AngWT complex.

The RMSF is defined as

(11) $${\mathrm{RMSF}}_{i}=\sqrt{\frac{\sum_{t=1}^{t=T}{\left(r_{it}-{\langle r_{i}\rangle }_{t}\right)}^{2}}{T}}$$

where, *t* indicates the snapshot or time slice in the simulation trajectory, $r_{it}$ is the position of atom *i* at time *t*, ${\langle r_{i}\rangle }_{t}$ is the average position of atom *i* during the simulation and *T* is the last snapshot considered. Therefore, this quantity provides a measure of the average distances that individual particles explore around their equilibrium positions. The difference between RMSD and RMSF is that while in the RMSD the average is performed on all the atoms in the protein for each snapshot or time slice in the simulation, in the RMSF the average is performed on each atom for all the snapshots of the simulation trajectory. The REST2 simulations showed that both the RMSD and RMSF of the Ang variants were always larger than those of the RNH1 inhibitor in all cases [38]. In addition, the RNH1 atoms do not suffer significant changes either in the RMSD or in the RMSF upon mutations in the Ang protein. Instead, the RMSD and RMSF of the Ang protein are indeed affected by the mutations (Table 1, Figure 3).

In particular, both RMSD and RMSF increase with regard to Ang in the RNH1-GGRR_mut_ complex, with respect to the RNH1-AngWT complex (Table 1, Figure 3), indicating that the mutated protein shows more flexibility and weaker interactions. This correlates with the experimental evidence that this mutant shows less affinity to the RNH1 inhibitor than AngWT. The QG mutant in complex with RNH1 exhibited lower RMSD and RMSF values than those of the complexed AngWT (Table 1, Figure 3), which indicates that this mutant establishes stronger interactions with RNH1 and correlates with the experimentally observed higher RNH1 affinity. Finally, the Ang protein in the RNH1-GGRR/QG_mut_ complex shows larger RMSD than that of the RNH1-AngWT complex but similar RMSF values (Table 1, Figure 3). The RMSD deviation indicates that the AngQG mutant binds in a different way than the RNH1-AngWT complex, but the similar RMSF values indicate that this different binding mode is stable, in agreement with the similar affinities for AngWT and AngQG measured experimentally.

Since RNH1 is up-regulated in a number of cancers, Ang-based hCFPs must comprise an Ang effector with reduced affinity for RNH1, such as by the introduction of large obstructing side chains at positions 85 and 86 (G85R and G86R). However, the above results suggest that enhancing Ang activity by synergistically reducing RNH1-Ang (mutant) interaction and increasing enzymatic activity may need to be achieved by a different combination of mutations with no distal effects.

## 8. Concluding Remarks

The combined experimental and computational studies on granzyme B and angiogenin proteins clearly show the power of the simulations in predicting the effect of mutations on the activity of corresponding targeted human cytolytic fusion proteins. On one hand, the inhibition susceptibility of the hCFPs can be improved by simulating the interaction of designed mutants with their protein inhibitors. For example, the simulations of RNH1 complexed with angiogenin variants or PI9 in the case of Granzyme B, gave a rationale for the experimental observations. On the other hand, the presented approaches also allow obtaining insights on the enzymatic activity. Here, the simulations on GrB gave indications that the proposed mutations would not negatively affect the catalytic activity. Indeed, the in vitro experiments, performed to test this hypothesis, showed that not only is the catalysis not worsened, but actually for the R201K variant is improved. This protocol is a model example of how of HPC-based simulations and state-of-the-art experiments can be combined as promising engineering tool for effective next generation therapeutics based on immunotoxins.

## Figures and Tables

**Table 1:** Root mean square deviation (RMSD) of the C_α_ carbons of WT angiogenin and the mutants QG_mut_, GGRR_mut_, GGRR/QG_mut_ complexed with RNH1 measured from REST2 simulations.

Variant	RMSD (Å)
WT	3.0 ± 0.6
QG_mut_	1.9 ± 0.4
GGRR_mut_	3.4 ± 0.8
GGRR/QG_mut_	4.2 ± 0.7

## Appendix A

### Appendix A.1. Molecular Docking

The aim of protein-protein docking is to predict the structure of a protein-protein complex. By starting from the unbound protein moieties the identification of the most likely binding conformations requires the exploration of a large number of different potential binding modes. Sampling of such large conformational space using molecular dynamics or Monte Carlo approaches, would be computationally impractical and very expensive even for a small-size system.

Several “protein-protein docking” algorithms can be preferably used, which in their simplest form are based on geometry considerations or shape complementarities. However, a major bottleneck of these methods is that they treat proteins as rigid-bodies [145]. Protein flexibility may be included to refine the rigid-body docking orientations. This approach is valid when there are only small conformational changes upon binding [44]. ICM-DISCO [146], RosettaDock [147] and HADDOCK [148,149] include such refinement steps after the rigid-body docking. HADDOCK is one of the very few docking programs that performs flexible docking, plus the possibility to include explicit water molecules. In addition, it requires experimental/theoretical restraints for the minimization. FireDock’s [150,151] docking refinement involves finding the optimal combination of side-chain rotamers with the lowest total energy, followed by rigid-body optimization and ranking of the new conformations. In cases in which binding is associated with large conformational changes, docking search programs should use scoring functions that account for such large conformational changes. ATTRACT [152] uses coarse-grained representation of the interacting molecules, and includes global flexibility by minimizing along the direction of pre-computed soft modes of the unbound proteins, i.e., collective modes corresponding to the eigenvectors with the smallest eigenvalues of the Hessian matrix of the potential energy function [153]. The program allows inclusion of conformational selection type mechanisms [154]. The SwarmDock program [155,156] uses a particle swarm optimization algorithm [157] to optimize the conformational degrees of freedom. Docking associated with very large conformational changes (like domain reorientations or transitions from disordered to structured conformations) are challenging, however promising solutions have been proposed [158].

The second part of each docking calculation is the evaluation of the binding pose using a scoring function; a fast mathematical tool to estimate the strength of the interactions between the proteins that have been docked.

It is possible to distinguish between four classes of scoring functions. These include:

- Force field based scoring functions that account large conformational changes by summing the strength of intermolecular van der Waals and electrostatic interactions between all atoms.
- Empirical scoring functions. Based on counting the number of various types of interactions between the two binding partners. Counting may be based, for example, on the number of atoms in the two proteins in contact with each other.
- Knowledge-based scoring functions (also known as statistical potentials). Based on statistical observations of intermolecular contacts in structural databases (e.g., the PDB). This approach assumes that atoms interacting with frequencies higher than those given by a random distribution are likely to be energetically more favorable.
- Machine learning scoring functions. The functional form for the relationship between the binding affinity and the structural features of the docked complex is inferred directly from the data. They have consistently been found to outperform classical scoring functions at predicting binding affinity of diverse protein-protein complexes [159].

### Appendix A.2. MD-Based Enhanced Sampling Methods

Enhanced sampling methods can be classified in a three main categories, according to their scope and range of applicability:

1. Collective-variable based methods. Free energy is calculated as a function of one or a few predefined collective variables (CVs), i.e., any differentiable function of the atomic coordinates associated to the degrees of freedom of the rare event under investigation in the system. Examples of these methods include thermodynamic integration [160], free energy perturbation [161], umbrella sampling [162], and weighted histogram techniques [163]. These approaches are very powerful but they do require a careful choice of the CVs that must provide a satisfactory description of the reaction coordinates. An alternative is offered by recently developed *non-equilibrium* methods; such as adaptive force bias [164], Wang-Landau sampling [165] and metadynamics [166]. In the latter approach, a history-dependent potential is added along selected CVs to compensate the underlying free energy landscape, allowing the system to escape from very stable minima and overcome high energy barriers. This way a reconstruction of the free energy profile along these variables can be performed. The application of the method is however limited to processes that can be described with a low number of CVs [167], constraining its applicability to biological events that are not high dimensional.
2. Path sampling methods are aimed at exploring the transition mechanism and constructing reactive trajectories, such as finite-temperature string method [168], transition path sampling [169] or transition interface sampling [170]. These approaches are extremely powerful but they can be applied only if one knows in advance the initial and final states of the process that has to be simulated. Yet, these approaches are very expensive computationally.
3. Generalized-ensemble approaches. These methods are based on non-Boltzmann probability weight factors so that a random walk in energy space may be realized [171,172]. This way such approaches allow the system to explore the phase space regardless the presence of high-energy barriers. In a single simulation it is possible to identify the energy minima and compute thermodynamic quantities in a wide temperature range. Two such well-known methods are the multi-canonical algorithm (random walk in energy space) [173] and the simulated tempering (random walk in temperature space) [174,175]. This approach is powerful but the probability weight factors that allow the random walk are not a priori known and usually are determined by several short trial simulations, a process usually laborious and not trivial. For this reason, an additional class of generalized-ensemble algorithms has been devised, the so-called replica-exchange methods (also referred to as replica Monte Carlo methods, multiple Markov chain method, and parallel tempering) [176], where the weight factor is essentially known and there is no complication in its determination. In practice, during these approaches the phase space is explored simultaneously by several copies of the system that differ for some specific properties (e.g., the temperature). Due to the replication approach, those methods are rather computational demanding and require HPC resources.
